# The translation and validation of the MES for an Austrian sample

**DOI:** 10.18332/ejm/191394

**Published:** 2024-09-09

**Authors:** Myriam N. Jordan, Antigoni Sarantaki, Athina Diamanti, Victoria Vivilaki

**Affiliations:** 1 Department of Midwifery, University of West Attica, Athens, Greece

**Keywords:** perinatal care, midwifery, silent empathy, compassionate care, being with the woman

## Abstract

**INTRODUCTION:**

Empathy plays an important role in midwifery care, not only for the women but also for midwives. The Midwifery Empathy Scale (MES) was developed to assess the empathy levels of midwives and midwifery students. The purpose of this study was the translation and validation of the MES for an Austrian sample.

**METHODS:**

A total of 277 midwives working in Austria completed the questionnaire of the MES. The psychometric measurements that were performed included explanatory factor analysis using a varimax rotation and principal components analysis. Moreover, the internal consistency of the MES was assessed with reliability coefficients. The Kaiser-Meyer-Olkin (KMO) measure of sampling adequacy and a Bartlett's test of sphericity were carried out.

**RESULTS:**

Principal components analysis showed seven orthogonal factors. KMO measure of sample adequacy = 0.724 and Bartlett’s test of sphericity = 1058.904 (df=231, p<0.0001). The MES showed an acceptable overall internal consistency: Cronbach’s alpha was found to be 0.721 and the Guttman split-half coefficient was 0.611. The findings of our study confirm the multidimensionality of MES, demonstrating a seven-factor structure which contained subscales reflecting empathy and emotional connection. The mean total score of Austrian midwives’ responses to the MES was 44.80 with scores ranging from 24 to 81.

**CONCLUSIONS:**

This study shows that the German version of the Midwifery Empathy Scale is a reliable instrument for evaluating the empathy levels of midwives and midwifery students in Austria. The German MES could be used in the selection and education of future midwives as well as in connection with empathy trainings of midwives.

## INTRODUCTION

Numerous studies have shown the importance of empathy in healthcare. Higher levels of empathy have been linked with better patient clinical outcomes^1^, higher patient satisfaction^2^, and more accurate diagnoses^3^. Moreover, there is evidence that high levels of empathy protect against development of burnout by healthcare professionals^4^.

When trying to define empathy, it becomes evident that there are several definitions. Rogers^5^ states that ‘the state of empathy, or being empathetic, is to perceive the internal frame of reference of another with accuracy, and with the emotional components and meaning which pertain thereto, as if one were the other person, but without ever losing the “as if” condition’.

Hojat et al.^6^ define empathy in the context of medical education and patient care as a mainly cognitive and not affective or emotional characteristic. Moreover, to be empathetic, the health care professional needs to understand instead of feel the patients’ experiences, concerns and perspectives and be able to communicate this understanding. Furthermore, there is the intention to help.

Hojat et al.^7^ also suggest that ‘physician empathy is a multidimensional concept involving at least three components. The most important component is perspective-taking, an outcome consistent with that reported for the general population. Other components of empathy are compassionate care and standing in the patient’s shoes, both specific to the patient-physician relationship’.

The International Confederation of Midwives^8^ states in the ‘Essential Competencies for Midwifery Practice’ that a midwife should ‘demonstrate effective interpersonal communication with women and families, health care teams, and community groups’ and needs to ‘listen to others in an unbiased and empathetic manner’.

Studies have shown the importance of empathetic midwifery care. Women being supported by midwives who are sensitive to their needs have increased satisfaction with their birth experiences^9^ and the hospital childbirth services^10^.

Nevertheless, studies with health professionals have shown decreasing empathy levels during the years of education and residency^6^. However, interventions for midwives that increase empathy can influence and improve mothers’ birth perception and satisfaction with midwives^11^. Because of this, Moloney and Gair^12^ find it important to teach and embed empathy in the curricula for midwifery students.

Although empathy is characterized as being difficult to measure^13^, Hojat et al.^14^ developed the Jefferson Scale of Empathy. The scale and its different versions were developed by Hojat et al.^14^ in 1999 and 2007 to assess the effectiveness of educational programs promoting empathy and can be used by physicians and other healthcare professionals. Numerous studies have used the Jefferson Scale of Empathy to assess empathy levels in different healthcare professionals^15,16^.

As Hogan et al.^17^ stated, the Jefferson Scale of Empathy is not ideal for midwifery; for instance, the word ‘patient’ should be replaced by a woman or client. Recently, Vivilaki et al.^18^ developed the Midwifery Empathy Scale, an instrument specifically for midwives and midwifery students. Until this point, it has been available in English and Greek.

The general aim of this study was to translate and validate this instrument into German. More specifically, the study’s objectives are to test a German version of the MES and assess its reliability and validity in identifying empathy levels in a sample of Austrian midwives and secondly to examine the factor structure of the German MES.

## METHODS

### First phase

*Translation of the original MES and pre-testing*

In this study, the World Health Organization’s guidelines for translation and adaptation of instruments were followed^19^. The permission to use and translate the MES was requested in written form and was granted by the authors. One translator with knowledge of the English-speaking culture but with a mother tongue of German, was given the task of forward translation. This translator was a health professional and familiar with the used terminology^19^. Additionally, unaware of the topic, one translator translated the questionnaire into his mother tongue (German). After the translation process, the two translators worked on and solved discrepancies between the two translations^20^. After forward translation, the questionnaire was independently back-translated. This was done by one translator with a mother tongue of English, unaware of the topic of the questionnaire^19^. The back-translation was given to the developers of the MES, and permission to work with the German version was granted.

After the translation of the MES from English to German, the instrument was pre-tested. Five respondents (midwives and midwifery students) were included in the pre-testing. As part of the cultural adaption process, a systematic debriefing was done with the respondents afterward, during which they were asked about the questionnaire questions. The respondents were asked if they had problems understanding the questions or single words. Furthermore, they were asked to explain why they answered in a certain way and how they answered. These answers were compared to the actual responses for consistency. Comments and suggestions made by the focus group were included in the final version^19^.

*Participants and data collection*

After pre-testing, the Austrian Midwives Association sent the final version to midwives living and working in Austria. Before sending out the questionnaire, it was reviewed by the Science Department of the Austrian Board of Midwives. The Austrian Midwives’ Association committee approved an online survey of Austrian midwives.

Along with the questionnaire, there was a cover letter explaining the purpose of the study, providing the researchers’ affiliation and contact information, and clearly stating that answers would be confidential and anonymity would be guaranteed in the final data reports.

The study participants were a sample of Austrian midwives. The inclusion criteria were working and living in Austria, being fluent in German, and having written informed consent. In total, 277 midwives agreed to participate.

### Instrument

Vivilaki et al.^18^ developed the Midwifery Empathy Scale in 2016, a 22-item psychometric scale specifically for midwives and midwifery students. Empathetic responses are measured with the help of 22 items. Every item is scored on a 6-point Likert scale: 1 = Totally Agree, 2 = Agree, 3 = Not Sure But Probably Agree, 4 = Not Sure But Probably Disagree, 5 = Disagree, and 6 = Totally Disagree. A total score is calculated, with the highest score of 132 (highest empathy) and lowest 22 (lowest empathy). Items measuring negative statements are reverse-scored. The MES is a reliable and valid instrument for evaluating the empathy levels of midwives and midwifery students.

### Statistical analysis

*Data analysis and validation*

Statistical analysis was performed with the help of IBM SPSS statistics version 23. Firstly, descriptive analysis was calculated for the MES items, including means with standard deviation, and frequencies and percentages. Internal consistency was measured by calculating Cronbach’s alpha and Guttman split-half coefficients. Factor analysis was conducted using principal components analysis.

*Reliability*

Cronbach’s alpha was carried out to assess the internal consistency of the instrument. The coefficient of Cronbach’s alpha should be >0.7 to fulfill the recommended level for new instruments^21^. Moreover, the internal consistency of the German MES was tested using Guttman split-half coefficients.

*Factor structure*

Explanatory factor analysis using a varimax rotation and principal components analysis was employed to explore the underlying dimensions of the MES scale. The following criteria were used to determine the dimensional structure of the MES: 1) eigenvalue >1^22^; 2) variables should load >0.40 on a factor^23^; 3) the interpretation of the factor structure should be meaningful; and 4) the Scree plot needs to be accurate when means of communalities are >0.40^24^. Computations were based on a covariance matrix, as all variables received values from the same measurement scale^25^. The Kaiser-Meyer-Olkin (KMO) measure of sampling adequacy and Bartlett’s test of sphericity were carried out to determine if the collected data were adequate for factor analysis^26^. As factor analysis found seven subscales, subsequent Cronbach’s alpha calculations were separately carried out for each subscale, highlighting how the items grouped.

*Face and content validity*

A research midwife investigated the meaning and acceptability of the items by the midwives during the scale administration.

## RESULTS

### Sample characteristics

**Figure 1:**
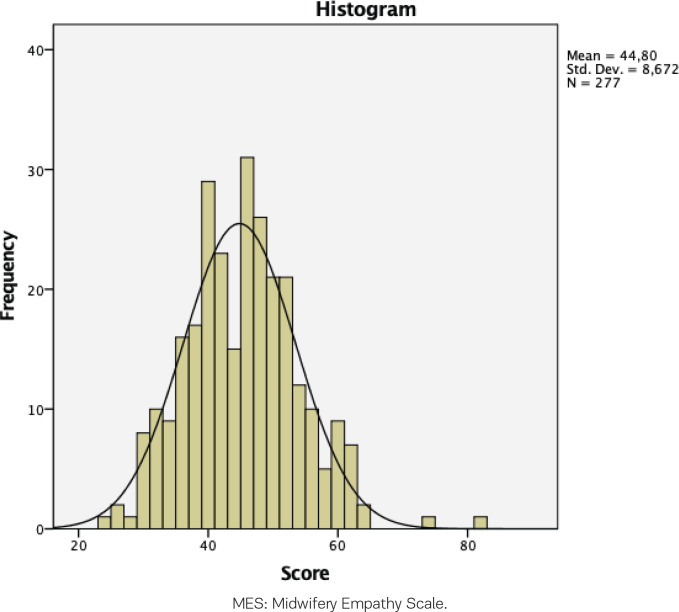
Frequency of total scores of the responses of Austrian midwives to the MES questions, December 2020 to March 2021 (N=277)

In December 2020, the final version of the translated questionnaire was sent to the Austrian Midwives Association, which agreed to forward the questionnaire to the midwives working in public and private hospitals and/or as independent midwives in Austria. From December 2020 to March 2021, a total of 277 filled-in questionnaires were sent back by the midwives. The final sample of 277 was suitable for exploratory factor analysis^27^. The total scores of the midwives ranged from 24 to 81 (maximum score possible 132, minimum score possible 22). The MES mean total score was 44.80. Figure 1 shows the frequencies of the midwives’ scores.

### Psychometric characteristics of MES

*Reliability*

The internal consistency characteristics of the German MES showed acceptable reliability. Cronbach’s alpha was 0.721 for the total scale (items 1–22), standardized alpha was 0.757, and Guttman split-half coefficient was 0.611.

*Factor structure*

Principal components analysis

**Figure 2:**
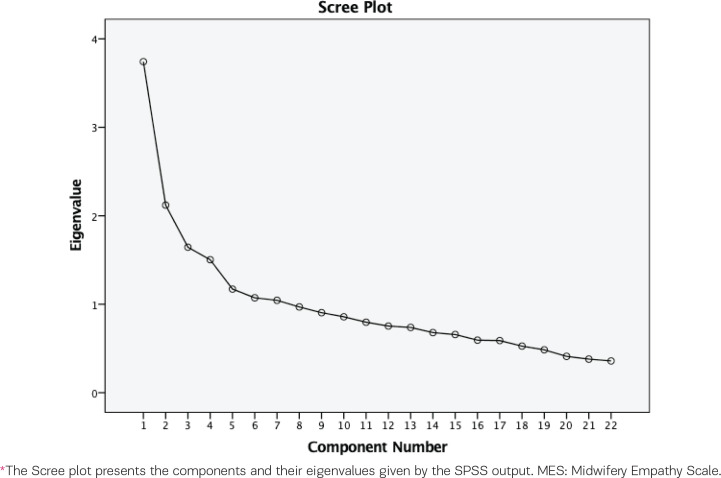
Scree plot* of the MES answered by Austrian midwives, December 2020 to March 2021 (N=277)

KMO measure of sampling adequacy was equal to 0.724, which implies a good sample size^28^. Bartlett’s test of sphericity was 1058.90 (df=231) and was highly significant (p<0.001), which indicates that the variables are correlated and, therefore, appropriate for PCA^29^. Figure 2 shows the Scree plot. Table 1 presents the descriptive statistics for the MES questions. Supplementary file Table 1 shows the frequencies for the MES items, Supplementary file Table 2 shows the exploratory factors and explained variance after rotation for the German MES, and Supplementary file Table 3 presents the communalities for the German MES.

**Table 1:** Mean scores* of the responses by Austrian midwives to the MES questions, December 2020 to March 2021 (N=277)

*Questions*	*Mean score*	*SD*
1. I believe that empathy plays an important role in midwifery care.	1.10	0.31
2. I can perceive the hidden feelings and thoughts of the women that are in my care.	1.99	0.69
3. Women feel better when they sense that they are understood.	1.12	0.32
4. I recognize the body language of a woman.	1.90	0.69
5. Body language is not as important as verbal communication for the understanding of the woman’s feelings.	4.91	1.11
6. I recognize when a woman is silent because of embarrassment.	2.21	0.88
7. I don’t get emotionally affected when I see women cry.	4.56	1.43
8. It is difficult for a midwife to see things from women’s perspective.	4.91	1.17
9. I try to stand in the woman’s shoes, so I can better understand her.	1.72	0.87
10. I show that I am willing to listen to the woman by always sitting near her.	2.39	1.15
11. I would spend time to take care of women after my work hours.	3.48	1.48
12. Midwife’s touch encourages the woman.	2.12	0.89
13. I avoid to touch the woman I am caring for, in order to keep a distance.	5.42	0.78
14. I think it is important to touch a woman when I am caring for her.	2.27	1.17
15. Very sensitive women irritate me.	5.27	1.00
16. There were times that I witnessed a woman cry and I got emotional.	2.07	1.25
17. Many times I left work and I kept thinking of a woman I was caring for.	1.87	1.12
18. I don’t think part of my job to occupy myself with the problems of the woman I care.	4.73	1.24
19. I feel satisfaction when women feel better with my care.	1.21	0.46
20. If I realize that a woman is afraid, I spend time trying to reassure her.	1.27	0.48
21. I could go against hospital rules in order to help a woman.	3.39	1.56
22. I usually stay emotionally detached from the women that are in my care.	4.52	1.28

MES: Midwifery Empathy Scale.

* Responses to questions are scored from 1 to 6. Responses to Questions 1, 2, 3, 4, 6, 9, 10, 11, 12, 14, 16, 17, 19, 20 and 21 are scored with totally agree = 6 to totally disagree = 1. Responses to Questions 5, 7, 8, 13, 15, 18 and 22 are reverse scored (totally agree = 1 to totally disagree = 6).

The PCA of the 22 items of the MES presented a seven-component solution. The eigenvalues were >1 for seven components, which explained 55.90 % of the data (Supplementary file Table 2).

The first factor (Silent Empathy) includes the following questions: 2, 3, 4, 6. The eigenvalue is 3.74, which explains 10.05% of the variance. The second factor (Midwife’s Touch) includes questions 12, 13, and 14; the eigenvalue is 2.12, and explains 9.20 % of the variance. The third factor (Being with Woman) includes questions 1, 10, and 20; the eigenvalue is 1.65, and explains 8.36% of the variance. The fourth factor (Emotional Connection) includes questions 7, 16, 17, and 19; the eigenvalue is 1.50 and explains 8.09% of the variance. The fifth factor (Sensitivity) includes questions 5, 8, and 15; the eigenvalue is 1.17, and explains 6.91% of the variance. The sixth factor (Perspective Taking) includes questions 9, 18, and 22; the eigenvalue is 1.07, and explains 6.88% of the variance. The seventh factor (Activism) includes questions 11 and 21; the eigenvalue is 1.04, and explains 6.42% of the variance.

*Validity*

Face and content validity

The midwives accepted the German version of the MES well. It was easily and quickly completed (approximately 10 minutes). The questions seemed to be relevant, reasonable, unambiguous, and clear. For that reason, face validity was considered to be very good. The German version of the MES includes, in a balanced way, the full range of the characteristics of empathy that it is intended to measure.

Construct validity

As mentioned above, the MES items were formed into seven subscales after using principal components analysis. Cronbach’s alpha was calculated for each of the subscales. Factor 1 (Silent Empathy: questions 2, 3, 4, 6) had a Cronbach’s alpha of 0.62. Factor 2 (Midwife’s Touch: questions 12, 13, 14) had 0.7, factor 3 (Being with Woman: questions 1, 10, 20) had 0.37, factor 4 (Emotional Connection: questions 7, 16, 17, 19) had 0.53, factor 5 (Sensitivity: questions 5, 8, 15) had 0.42, factor 6 (Perspective Taking: questions 9, 18, 22) had 0.45 and factor 7 (Activism: questions 11 and 21) had 0.47.

## DISCUSSION

### Main findings

This study aimed to translate and validate the MES for a German-speaking sample. The MES was developed by Vivilaki et al.^18^ in order to have a psychometric tool that measures empathy levels in midwives and midwifery students. The total scores of the Austrian midwives ranged from 24 to 81 (maximum score possible 132, minimum score possible 22). The mean MES total score was 44.80. The Kaiser-Meyer-Olkin measure of sampling adequacy (0.72) and Bartlett’s test of sphericity (p<0.001) confirmed that the collected data were adequate for factor analysis. Factor analysis was performed using the principal components analysis and varimax rotation. The eigenvalues were >1 for seven factors, explaining 55.90% of the variance. Cronbach’s alpha was carried out for each of the seven subscales identified by factor analysis. Cronbach’s alpha was 0.62 for the first subscale, 0.7 for the second, 0.37 for the third, 0.53 for the fourth, 0.42 for the fifth, 0.45 for the sixth, and 0.47 for the seventh. According to this study, the major formative factors of the empathy levels in Austrian midwives are: 1) Silent Empathy, 2) Midwife’s Touch, 3) Being with Woman, 4) Emotional Connection, 5) Sensitivity, 6) Perspective Taking, and 7) Activism.

Bradfield et al.^30^ state that the idea of being ‘with woman’ (factor 3) is a central construct of the profession of midwives. Their findings show that ‘midwives who were not displaying the characteristics and manifestations of the phenomenon were described as not “doing” midwifery, or not “being” midwives but merely persons providing care’. According to Bradfield et al.^31^, the concept of being ‘with woman’ is a part of different international standards and publications of midwifery associations.

Factor analysis showed the multidimensionality of the MES for an Austrian sample, showing a seven-factor structure. Cronbach standardized alpha for the German MES was found to be higher than the one reported by Vivilaki et al.^18^ (0.55). In comparison to the results of the Greek MES^18^ (Factor 1 ‘Compassionate Care’ explaining 24.63% of the variance), factor 1 for the Austrian sample was ‘Silent Empathy’.

Overall, European midwives share common cultural characteristics, such as general midwifery values and principles that the European midwives share. However, the local cultural differences and the divergent educational programs in the European member states^32^ result in different perceptions, highlighted in factor analysis. As a result, this is an important challenge – in terms of empathy – that midwives could face if trained in one country and have to adapt their midwifery practice in another culturally.

Several studies have shown a decline in empathy during medical school and residency^3,6^. According to Hojat et al.^6^, the decline of empathy has many different reasons, ‘including lack of role models, a high volume of materials to learn, time pressure, and patient and environmental factors’. Studies on the decline or increase of empathy in midwifery students are rare and should be addressed more in the future. As far as the authors know, only two published studies evaluate midwifery students and their empathy levels over time^33,34^. According to McKenna et al.^33^, the mean empathy scores of the assessed 52 undergraduate midwifery students were lower than empathy scores from studies with other health professionals. However, contrary to the studies stated above, the students’ empathy scores were lowest in the first year and increased consistently with every year of the Bachelor’s Program. The second study conducted with midwifery students showed no statistically significant trend of declining empathy scores^34^. It would be interesting to investigate the reasons for the increase in empathy levels in the study of McKenna et al.^33^ as it could help other universities arrange curriculum content.

Studies on empathy training for midwifery students have shown that interventions can increase empathy levels in students. These increases can be seen immediately after the intervention and additionally after some time at the follow-up test^17^.

The validated MES could be reliable for evaluating the empathy levels of midwives and midwifery students in Austria. One possible field of application could be the annual entrance examination for undergraduate midwifery courses, as the importance of high empathy levels in future midwives is evident. Moreover, the MES could be used before and after interventions that increase empathy levels, particularly training for students and midwives.

### Limitations

This study was not without limitations. Due to the pandemic, the questionnaire was sent out by an online survey tool without in-depth interviews, which may have resulted in better-investigating empathy. Despite the above limitation, this study investigates the empathy levels of Austrian midwives. Another limitation was that the authors did not use a questionnaire assessing the patient’s perception of the midwife’s empathy, such as the German version of the Consultation and Relational Empathy (CARE)^35^, for evaluating the empathy levels of midwives participating in this study. Furthermore, the authors could have investigated if the questionnaire results were consistent over time by checking the test-retest reliability of the scale over a short time. Participants represented the Austrian midwives regardless of the small targeted population and sample size. Rapid socioeconomic changes over the last few years have led to a relatively homogenous cultural background between Austrian midwives and midwives from other German-speaking countries. Despite the above concerns, our sample size is considered excellent for explanatory factor analysis. Our findings confirm the multidimensionality of the MES, demonstrating a seven-factor structure, while the sub-scales of the German MES showed good values for Cronbach’s alpha. The varied cultural backgrounds of our study population may explain significant differences in item factor loading characteristics. It is evident to the authors that further investigations on the strengths and weaknesses of the questionnaire are needed. Nevertheless, we believe the questionnaire can be useful for midwives and midwifery students in Austria. The implications for midwifery practice are better patient clinical outcomes, higher patient satisfaction, more accurate diagnoses, and a prevention strategy against burnout development of healthcare professionals.

## CONCLUSIONS

The aim of this study was to translate and validate the MES for an Austrian sample. A total of 277 midwives working in Austria completed the questionnaire, and the results showed satisfactory reliability. With the help of principal components analysis, explanatory factor analysis determined seven MES subscales. Therefore, we believe that it is a reliable and valid tool for identifying empathy levels, and midwife educators and managers can use it to improve the assessment and education of midwives and midwifery students.

## Supplementary Material



## Data Availability

The data supporting this research are available from the authors on reasonable request.
