# Supplementary material for: The translation and validation of the MES for an Austrian sample
Source: Eur J Midwifery. 2024 Sep 9;8:10.18332/ejm/191394. doi: 10.18332/ejm/191394 (PMC11440047; doi:10.18332/ejm/191394)
Supplement: Supplementary file 1 [file EJM-8-54-s1.pdf]

Supplementary Online Material

Table 1

Frequencies and percent of the answers given by the Austrian midwives for every item of the questionnaire (=22).

		Frequency	Percent	Cumulative Percent
**MES1: I believe that empathy plays an important role in midwifery care.**	totally agree	251	90.6	90.6
	agree	25	9.0	99.6
	not sure but probably agree	1	0.4	100.0
**MES2: I can perceive the hidden feelings and thoughts of the women that are in my care.**	totally agree	66	23.8	23.8
	agree	149	53.8	77.6
	not sure but probably agree	61	22.0	99.6
	not sure but probably disagree	1	0.4	100.0
**MES3: Women feel better when they sense that they are understood.**	totally agree	245	88.4	88.4
	agree	32	11.6	100.0
**MES4: I recognize the body language of a woman.**	totally agree	79	28.5	28.5
	agree	147	53.1	81.6
	not sure but probably agree	50	18.1	99.6
	not sure but probably disagree	1	0.4	100.0
**MES5: Body language is not as important as verbal communication for the understanding of the woman's feelings.**	totally agree	5	1.8	1.8
	agree	12	4.3	6.1
	not sure but probably agree	9	3.2	9.4
	not sure but probably disagree	33	11.9	21.3
	disagree	135	48.7	70.0
	totally disagree	83	30.0	100.0
**MES6: I recognize when a woman is silent because of embarrassment.**	totally agree	63	22.7	22.7
	agree	113	40.8	63.5
	not sure but probably agree	83	30.0	93.5
	not sure but probably disagree	17	6.1	99.6
	disagree	1	0.4	100.0
**MES7: I don't get emotionally affected when I see women cry.**	totally agree	11	4.0	4.0
	agree	20	7.2	11.2
	not sure but probably agree	35	12.6	23.8

	not sure but probably disagree	34	12.3	36.1
	disagree	91	32.9	69.0
	totally disagree	86	31.0	100.0
**MES8: It is difficult for a midwife to see things from womens' perspective.**	totally agree	6	2.2	2.2
	agree	7	2.5	4.7
	not sure but probably agree	24	8.7	13.4
	not sure but probably disagree	28	10.1	23.5
	disagree	117	42.2	65.7
	totally disagree	95	34.3	100.0
**MES9: I try to stand in the woman's shoes, so I can better understand her.**	totally agree	131	47.3	47.3
	agree	110	39.7	87.0
	not sure but probably agree	25	9.0	96.0
	not sure but probably disagree	6	2.2	98.2
	disagree	4	1.4	99.6
	totally disagree	1	0.4	100.0
**MES10: I show that I am willing to listen to the woman by always sitting near her.**	totally agree	66	23.8	23.8
	agree	101	36.5	60.3
	not sure but probably agree	64	23.1	83.4
	not sure but probably disagree	33	11.9	95.3
	disagree	9	3.2	98.6
	totally disagree	4	1.4	100.0
**MES11: I would spend time to take care of women after my work hours.**	totally agree	31	11.2	11.2
	agree	46	16.6	27.8
	not sure but probably agree	65	23.5	51.3
	not sure but probably disagree	50	18.1	69.3
	disagree	64	23.1	92.4
	totally disagree	21	7.6	100.0
**MES12: Midwife's touch encourages the woman.**	totally agree	71	25.6	25.6
	agree	120	43.3	69.0
	not sure but probably agree	71	25.6	94.6
	not sure but probably disagree	11	4.0	98.6
	disagree	4	1.4	100.0
	agree	2	0.7	0.7

**MES13: I avoid to touch the woman I am caring for, in order to keep a distance.**	not sure but probably agree	8	2.9	3.6
	not sure but probably disagree	15	5.4	9.0
	disagree	100	36.1	45.1
	totally disagree	152	54.9	100.0
**MES14: I think it is important to touch a woman when I am caring for her.**	totally agree	74	26.7	26.7
	agree	109	39.4	66.1
	not sure but probably agree	62	22.4	88.4
	not sure but probably disagree	15	5.4	93.9
	disagree	10	3.6	97,5
	totally disagree	7	2.5	100,0
**MES15: Very sensitive women irritate me.**	totally agree	2	0.7	0,7
	agree	4	1.4	2,2
	not sure but probably agree	17	6.1	8,3
	not sure but probably disagree	17	6.1	14,4
	disagree	92	33.2	47,7
	totally disagree	145	52.3	100,0
**MES16: There were times that I witnessed a woman cry and I got emotional.**	totally agree	111	40.1	40,1
	agree	97	35.0	75,1
	not sure but probably agree	35	12.6	87,7
	not sure but probably disagree	11	4.0	91,7
	disagree	18	6.5	98,2
	totally disagree	5	1.8	100,0
**MES17: Many times I left work and I kept thinking of a woman I was caring for.**	totally agree	135	48.7	48,7
	agree	85	30.7	79,4
	not sure but probably agree	30	10.8	90,3
	not sure but probably disagree	16	5.8	96,0
	disagree	8	2.9	98,9
	totally disagree	3	1.1	100,0
**MES18: I don't think part of my job to occupy myself with the problems of the woman I care.**	totally agree	6	2.2	2,2
	agree	10	3.6	5,8
	not sure but probably agree	31	11.2	17,0
	not sure but probably disagree	46	16.6	33,6
	disagree	98	35.4	69,0

	totally disagree	86	31.0	100,0
**MES19: I feel satisfaction when women feel better with my care.**	totally agree	225	81.2	81.2
	agree	48	17.3	98.6
	not sure but probably agree	3	1.1	99.6
	not sure but probably disagree	1	0.4	100.0
**MES20: If I realize that a woman is afraid, I spend time trying to reassure her.**	totally agree	205	74.0	74.0
	agree	68	24.5	98.6
	not sure but probably agree	4	1.4	100.0
**MES21: I could go against hospital rules in order to help a woman.**	totally agree	36	13.0	13.0
	agree	54	19.5	32.5
	not sure but probably agree	63	22.7	55.2
	not sure but probably disagree	44	15.9	71.1
	disagree	49	17.7	88.8
	totally disagree	31	11.2	100.0
**MES22: I usually stay emotionally detached from the women that are in my care.**	totally agree	9	3.2	3.2
	agree	12	4.3	7.6
	not sure but probably agree	33	11.9	19.5
	not sure but probably disagree	64	23.1	42.6
	disagree	91	32.9	75.5
	totally disagree	68	24.5	100.0

*MES: Midwifery Empathy Scale

Table 2

Exploratory factors and Explained Variance after rotation for MES

Factors		Rescaled Loadings	Eigen values	Rotation Sums of Squared Loadings			
				% of Variance	Cumulative Variance	Cronbach's alpha	Standardised alpha
Factor 1 **(Silent Empathy)**	Q2	0.666	3.742	10.045	10.045	0.620	0.623
	Q3	0.492					
	Q4	0.669					
	Q6	0.720					
Factor 2 **(Midwife's Touch)**	Q12	0.804	2.120	9.201	19.246	0.699	0.708
	Q13	0.668					
	Q14	0.820					
Factor 3 **(Being with Woman)**	Q1	0.669	1.645	8.361	27.607	0.367	0.493
	Q10	0.537					
	Q20	0.649					
Factor 4 **(Emotional Connection)**	Q7	0.691	1.503	8.085	35.692	0.532	0.550
	Q16	0.649					
	Q17	0.594					
	Q19	0.450					
Factor 5 **(Sensitivity)**	Q5	0.536	1.172	6.914	42.606	0.416	0.417
	Q8	0.617					
	Q15	0.693					
Factor 6 **(Perspective Taking)**	Q9	0.443	1.073	6.880	49.486	0.453	0.451
	Q18	0.688					
	Q22	0.561					
Factor 7 **(Activism)**	Q11	0.603	1.044	6.417	55.903	0.473	0.474
	Q21	0.833					

*MES: Midwifery Empathy Scale, Q: Question
Explanatory factor analysis using a Varimax rotation and Principal Components Method of the 22 items of the MES presented a seven-component solution. Table 2 shows that the eigenvalues were >1 for seven components. Furthermore table 2 presents the percentage of variance, Cronbach's alpha and standardized alpha for each factor.

Table 3

Communalities of the MES after applying the extraction method of Principal Component Analysis

	Initial	Extraction
MES1	1,000	0.513
MES2	1,000	0.499
MES3	1,000	0.423
MES4	1,000	0.516
MES6	1,000	0.574
MES9	1,000	0.416
MES10	1,000	0.516
MES11	1,000	0.551
MES12	1,000	0.715
MES14	1,000	0.736
MES16	1,000	0.624
MES17	1,000	0.514
MES19	1,000	0.513
MES20	1,000	0.600
MES21	1,000	0.734
MES5_r	1,000	0.475
MES7_r	1,000	0.503
MES8_r	1,000	0.532
MES13_r	1,000	0.567
MES15_r	1,000	0.661
MES18_r	1,000	0.575
MES22_r	1,000	0.543

*MES: Midwifery Empathy Scale, r: reverse coded
Table 3 presents the communalities of the German MES given by the SPSS output. The scree plot was considered accurate when means of communalities were above 0.40.

© 2024 Jordan M.N. et al.
